# 

**Published:** 1905-01-15

**Authors:** 


### CONTENTS.

Event and Comment - - - - - - - - - - 1
French Requirements and the D.D.S. in France,
By Chas. J. Gray, D.D.S. 9
Somnoform and its Administration - - - - - - - 16
Somnoform; Its Physiological Action and Its Administration,
By Dr. Florestan Aguilar 19
Dental Poverty, - - - - By G. S. Junkerman, D.D.S. 29
Army Dental Corps, - - - By J. S. Marshall, M.D., D.D.S. 37
A Few Facts About Dental Caries,
By Dr. Aug. Lohmann and Dr. E. C. Kirk. 42
Announcements - - - - - - - - - - 45
Bibliography - - - - - - - - - - - 46
Indents - - - - - - - - - - - 49

